# Statistics on Cannabis Users Skew Perceptions of Cannabis Use

**DOI:** 10.3389/fpsyt.2013.00138

**Published:** 2013-11-06

**Authors:** Rachel M. Burns, Jonathan P. Caulkins, Susan S. Everingham, Beau Kilmer

**Affiliations:** ^1^RAND Corporation, Drug Policy Research Center, Pittsburgh, PA, USA; ^2^Carnegie Mellon University, Heinz College, Pittsburgh, PA, USA; ^3^RAND Corporation, Drug Policy Research Center, Santa Monica, CA, USA

**Keywords:** cannabis, marijuana, substance abuse research, drug use metrics, drug use trends

## Abstract

Collecting information about the prevalence of cannabis use is necessary but not sufficient for understanding the size, dynamics, and outcomes associated with cannabis markets. This paper uses two data sets describing cannabis consumption in the United States and Europe to highlight (1) differences in inferences about sub-populations based on the measure used to quantify cannabis-related activity; (2) how different measures of cannabis-related activity can be used to more accurately describe trends in cannabis usage over time; and (3) the correlation between frequency of use in the past-month and average grams consumed per use-day. Key findings: focusing on days of use instead of prevalence shows substantially greater increases in U.S. cannabis use in recent years; however, the recent increase is mostly among adults, not youth. Relatively more rapid growth in use days also occurred among the college-educated and Hispanics. Further, data from a survey conducted in seven European countries show a strong positive correlation between frequency of use and quantity consumed per day of use, suggesting consumption is even more skewed toward the minority of heavy users than is suggested by days-of-use calculations.

## Introduction

In substance abuse research, “use” is operationalized in terms of prevalence (i.e., how many individuals used a drug within a given period of time). However, prevalence is neither the only nor the ideal metric available. Other metrics, such as quantity of drug consumed may provide more insights into behaviors associated with intoxication and health-related outcomes, contact with law enforcement, and flows of money into black markets.

Studying users is perhaps the norm in substance abuse epidemiological research. One can ask a sample of people (e.g., in households or students in classrooms) questions about their drug use in order to learn, for example, how many used a given drug in the past-year, and on how many days did they consume. This is undoubtedly a useful perspective. We might find out, for example, that most marijuana users did not purchase the marijuana they consumed most recently; instead, it was shared with them or given to them for free.

However, imagine we could instead sample on the drug or, equivalently, the episode of drug use, rather than on the user. That would be like taking a random sample of all the grams consumed over the past-year, and asking: what are the users of this drug like? As we report below, from that perspective 88% of marijuana is consumed by someone who most recently obtained marijuana by purchasing it (as opposed to sharing or receiving it as a gift). The two perspectives suggest very different conclusions concerning the relative importance of purchases vs. gifts in retail marijuana distribution.

If the goal is to understand the drug-using careers of users, we might prefer to study a sample of users. But if the goal is to understand market-related quantities like how demand is affected by price or the roots of systemic violence, following the drug could be more valuable.

Naturally it is not literally possible to sample on chunks of the drug. No one assigns each gram a unique identification number, let alone a phone number that survey researchers could call. However, we can approximate this by weighting respondents by the quantities they consume. The purpose of this paper is to use a variety of data sets describing cannabis consumption to highlight the sometimes substantial differences in inference that arise when focusing on consumption, not consumers.

Before proceeding let us illustrate the principle numerically with a simple, extreme, and stylized example. Suppose there are just two kinds of cannabis users, “light” and “heavy,” who use 1 g and 1 ounce per month, respectively. Suppose further that 80% of users are light users, so there are four light users for every heavy user. Obviously when sampling on users, one would report that most cannabis users are light, few are heavy.

But since each heavy user consumes about 28 times as much as a light user (1 ounce = 28.35 g), the heavy users consume 28/(28 + 4 × 1) = 88% of the cannabis. Prior research has shown that different conclusions can be drawn when observing light vs. heavy users [e.g., (1)].

Furthermore, if over time there were no change in the number of cannabis users, but the ratio of light vs. heavy users switched from 80/20 to 20/80, then consumption would increase by 250% even though there was no change whatsoever in the number of users.

Because there is actually a continuum of usage, the difference between studying cannabis users and studying cannabis use is not so extreme, but it is large enough to matter, as we demonstrate below with a variety of examples. The basic observation is that when a covariate is positively correlated with quantity consumed conditional on there being some use, then individuals with that covariate account for a greater share of use than they do of users. For example, male users consume more than female users, so males account for a larger share of consumption than they do of prevalence. Conversely, users who are college graduates consume less intensively than do less educated users, so college graduates account for a smaller share of cannabis consumption than they do of cannabis users.

## Data

The National Survey on Drug Use and Health (NSDUH) is a nationally representative survey consisting of interviews conducted with randomly selected individuals ages 12 and older. NSDUH contains data on the prevalence of the use and abuse of alcohol, tobacco, and illegal substances. The survey contains sample weights that were used for all analyses to provide national-level estimates. From 2002 through 2011, there is an annual series of comparable data on past-year and past-30-day cannabis use (which we will refer to as “past-month use” throughout this paper), the number of use-days in the past-month for those who used in the last month, the number of use-days in the past-year for others who used in the last year, whether cannabis was purchased in the last month, the number of purchases in the last month for those who bought cannabis in the last month, and information about the most recent purchase of cannabis (amount purchased, cost of purchase, location of purchase, etc.). Starting in 2004 NSDUH also contains information about use of blunts (hollowed out cigar shells filled with cannabis), which was used to refine counts of past-month cannabis users and use-days for those years the item was available. While the impact of including survey items about blunts is small, it should be noted that counts of cannabis users and use-days from 2002 to 2003 may be slightly underestimated. In addition, NSDUH contains demographic information for each respondent that can be used to characterize users. One limitation of NSDUH is that it relies on self-report, which may introduce social desirability or recall bias (2). Another limitation of NSDUH is that it does not collect data from some populations that are known to have higher rates of illicit drug use, such as the incarcerated and homeless who are not in shelters (SAMHSA), but this can be shown to be a relatively insignificant deficiency in the case of cannabis (3).

The EU Drugs Markets II (EUMII) web-survey conducted by van Laar et al. (4) gathered information from a convenience sample of 4,156 cannabis users in seven countries: Bulgaria (*n* = 208), the Czech Republic (522), Italy (1,104), the Netherlands (1,128), Portugal (150), Sweden (791), and the United Kingdom (283). We focus on 2,530 observations since 1,626 of the respondents did not sufficiently answer the questions about quantity consumed (days per month, units per day, and grams per unit). For additional analyses of the EUMII cannabis data, see Caulkins et al. (5). As survey respondents often have difficulty answering directly questions about quantity consumed per day, this survey’s great innovation was to present respondents with picture cards, visually contrasting various amounts of cannabis with both a ruler and a credit card, to facilitate their ability to estimate how much they have consumed.

The EUMII survey has a number of limitations. Since it is an internet survey based on a convenience sample largely recruited on the web (i.e., no sampling frame), there is an obvious selection bias toward those who (1) use the internet, (2) are not concerned with sharing data about illegal behaviors online, and (3) think that volunteering to complete marijuana surveys is a good use of their time. van Laar et al. (4) report that while internet penetration in the EU is high (72% of the population), there was variation among the selected countries – 49% in Bulgaria, 51% in Portugal, 58% in Italy, 71% in the Czech Republic, 84% in the United Kingdom, 90% in the Netherlands, and 92.9% in Sweden (Internet World Stats 2011). Furthermore, recruitment methods differed by country and van Laar et al. note that most countries employed strategies that biased the sample toward attracting students and young adults.

Also some respondents may try to complete the survey multiple times or give unrealistic answers. Since incentives were not offered to complete the survey, we are less concerned about the former. As for the latter, which is not unique to web surveys, van Laar et al. (4) screened the data, setting unrealistic values to missing and dropping respondents “who indicated consuming more than 20 units (joints, pipes etc.) on an average use day” (68).

These limitations preclude using the EUMII data for estimating relative numbers of low- vs. high-frequency users, or for contrasting patterns across countries, but they should be of less concern when using the data as we do here to explore the correlation between use-days in the past-month and the average number of joints consumed per use day, particularly since the results are consistent with analyses from the U.S. (6) and Canada (7).

## Methods and Results

In the next three sections, we highlight (1) differences in inferences about sub-populations based on the measure used to quantify cannabis-related activity (past-year use, past-month use, past-month days of use, any past-month purchase, and number of past-month purchases); (2) how different measures can be employed to describe more accurately trends in cannabis usage over time; and (3) the correlation between frequency of use in the past-month and average grams consumed per day.

### Cross-sectional comparisons of use vs. use-days in NSDUH

National survey on drug use and health asks respondents whether they used cannabis in the last year, whether they used in the last month and, if so, how many days they used within the last month. It also asks whether they bought in the last month and, if so, how often. For any given subpopulation, say males, these variables let one define five proportions:

1. Males’ share of past-year users.
2. Males’ share of past-month users.
3. Males’ share of past-month days of use.
4. Males’ share of those who purchased within the last month.
5. Males’ share of past-month purchases.

Sometimes the proportions are all very close. Often they vary, sometimes substantially. As a general rule, for any attribute that is positively associated with cannabis use, the strength of that association grows as one moves through the list of the five proportions. For example, males use more cannabis than females. That is apparent even in simple past-year prevalence; males account for 60% of past-year cannabis users identified by the 2011 NSDUH. That proportion grows to 64% of past-month users, 69% of past-month days of use, 70% of past-month purchasers, and 72% of past-month purchases. Table 1 shows these five proportions for a variety of groups.

**Table 1:** **Various populations’ shares of cannabis-related activity by five different measures of participation, 2011 NSDUH**.

	Past-year users (%)	Past-month users (%)	Past-month days of use (%)	Past-month purchasers (%)	Past-month purchases (%)
Risk factors
Males	60^a^	64	69^a^	70^a^	72^a^
Used an illegal drug other than cannabis in
Past-year	35^a^	40	47^a^	46^a^	51^a^
Past-month	15^a^	20	24^a^	24^a^	29^a^
Past-month use of
Cocaine	4	5	7^a^	6	7^a^
Cigarettes	53^a^	59	66^a^	67^a^	76^a^
Alcohol	77^a^	80	79	81	81
Blunts	27^a^	42	54^a^	57^a^	73
Met criteria in past-year for
Cannabis dependence	9^a^	12	18^a^	19^a^	27^a^
Cannabis abuse or dependence	14^a^	19	26^a^	26^a^	37^a^
Abuse of dependence, any substance	33^a^	37	42^a^	43^a^	52^a^
Bought cannabis used last time	45^a^	56	70^a^	85^a^	88^a^
Ever arrested and booked	36^a^	40	47^a^	47^a^	53^a^
Drove under influence of drugs (past-year)	29^a^	37	47^a^	46^a^	47^a^
Adult with less than high school education	14	16	19	19	24^a^
Protective factors
College graduate	19	17	13^a^	11^a^	5^a^
Married	22	21	21	19	13^a^
Family income > $75,000	25	23	20	20	15^a^

^a^Indicates that proportion is statistically significantly different from proportion of past-month users (*p* < 0.05).

Variation across some rows is striking. Only 14% of past-year cannabis users meet the criteria for cannabis abuse or dependence, but they account for 26% of past-month days of use and 37% of past-month purchases. Perhaps the most striking contrast concerns blunts. Only 27% of past-year cannabis users report using a blunt within the last month, but those individuals account for 73% of cannabis purchases. On the protective factors side, the affluent, married, and college grads tend to use moderately; for example, college graduates account for 19% of past-year users, but only 13% of days of use and just 5% of purchases.

There is literature examining disparities in criminal justice sanctioning of drug users [e.g., (8–10)]. With varying degrees of sophistication, these studies compare for given groups (e.g., African-Americans) the share of some measure of sanctioning (arrests, convictions, incarceration, etc.) with their share of use or a use-related proxy. If the groups’ shares of all use-related measure were the same, then it would not matter much which measure was used. But figures for those who were ever arrested and booked (see Table 1) show that is not the case. Hence, to get a more complete picture of disparities, such studies should probably do the comparison with the full range of measures considered in Table 1. This is not a novel idea; Brownsberger (11) noted something similar with respect to alternate measures of crack use. But it is important.

**Figure 1:**
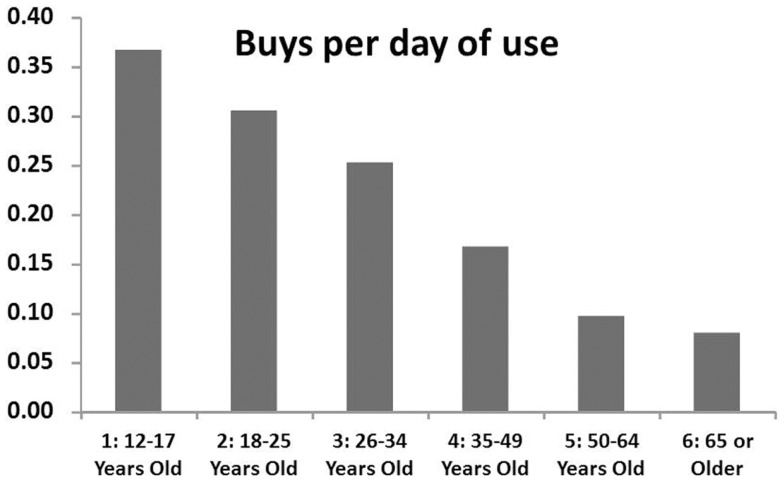
**The reported number of purchases per day of use varies dramatically with age in U.S. household survey data, 2011 NSDUH**.

To give one example, consider the distinction between using and purchasing. Possession and use *per se* carry relatively little risk of arrest. As Nguyen and Reuter (10) show, the probability of arrest per episode of cannabis use in the United States is only about 1 in 3,000. Purchasing by contrast may carry a greater risk of arrest, although there is some question about the proportion of drug arrests attributable to purchase transactions (12). If the number of purchases per day of use were the same across all groups, this would be a distinction without a difference. However, as Figure 1 shows, young people collectively report making more purchases per day of reported use than do older users. For example, 12–17-year-olds report fewer past-month days of use than do 50–64-year-olds (21 vs. 33 million), but many more past-month purchases (7.6 vs. 3.2 million).

Statistics indicating that the burden of arrest falls disproportionately on youth relative to their share of all *users* (9) may not be *prima facie* evidence of discrimination if making more purchases per day of use increases the risk of arrest per year of use. For example, 18–25-year-olds account for 49% of NSDUH respondents reporting having been arrested for a drug offense, even though they account for only 35% of past-year cannabis users. That appears to be a disproportionate arrest burden, but 18–25-year-olds account for 46% of buys reported in the past-month.

Likewise, the rate of arrest among past-year adult cannabis users is considerably higher for those with less than a high school education than overall (3.6 vs. 2.5 arrests per 100 users), but they make more buys per day of use, so the number of arrests per 100 buys is actually slightly below average (1.1 vs. 1.2 for adults overall).

Table 2 illustrates patterns for race and educational status. Non-Hispanic blacks represent 13% of past-year cannabis users vs. 23% of drug arrests reported by those users, but they report making 24% of the buys. Thus, some of their higher arrest rate may be a consequence of their purchase patterns. Indeed, Ramchand et al. (13) suggest that African-Americans may not only make more buys but also make riskier buys (e.g., more likely to buy outdoors).

**Table 2:** **Past-year use, number of drug-related arrests, and number of monthly purchases by education level and racial-ethnic group, 2011 NSDUH**.

	Proportion of past-year users (%)	Proportion of those arrested for drug offenses (%)	Proportion of buys (%)
**(AMONG ADULTS)**			
Less than high school	16	23	27
High school graduate	31	39	37
Some college	32	34	30
College graduate	31	3	6
**(AMONG ALL USERS)**			
Non-Hispanic white	67	53	55
Non-Hispanic black	13	23	24
Hispanic	14	18	15
Other	6	7	6

In sum, the measure of use matters. Therefore when drawing inferences about use one should consider which measure of use is appropriate in any given context and/or test to see if the conclusions are robust with respect to the measure of use employed.

### Comparing use vs. use-days in NSDUH over time

The analysis above pertains to snap-shots based on the 2011 NSDUH, yet trends over time provide another interesting perspective. In this section, we explore trends in cannabis use from years 2002 through 2011 of NSDUH and show how studying past-month use-days provides additional information about changes in cannabis usage not apparent when merely studying prevalence of use.

**Figure 2:**
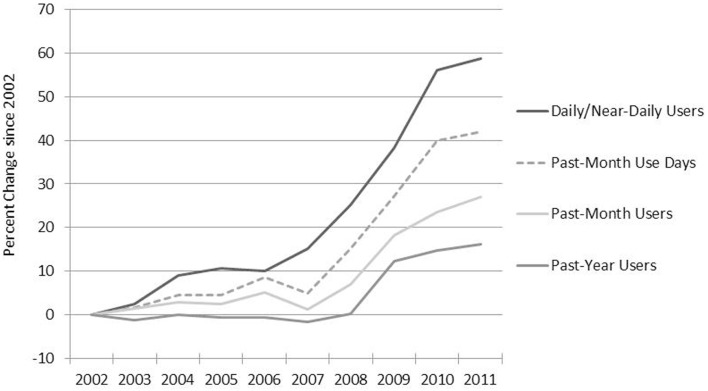
**Past-month use-days and daily near-daily users increased more rapidly from 2002 to 2011 than past-year and past-month users, NSDUH**. Note: because NSDUH did not collect data about blunts in 2002 and 2003, use-days may be underestimated for these years.

Figure 2 shows the change since 2002 in four measures of cannabis use: past-year prevalence, past-month prevalence (past-month users), number of daily/near-daily users (those who used cannabis 21 days or more in the last month), and past-month days of use. All four measures show an increasing trend, but the growth in usage (proxied by past-month use-days) outstrips the growth in consumers because of the increase in daily/near-daily use. That is, consumption grew primarily because of an increase in the average frequency of use, not just because of an increase in the overall number of users.

#### Proportion who are daily/near-daily users

**Figure 3:**
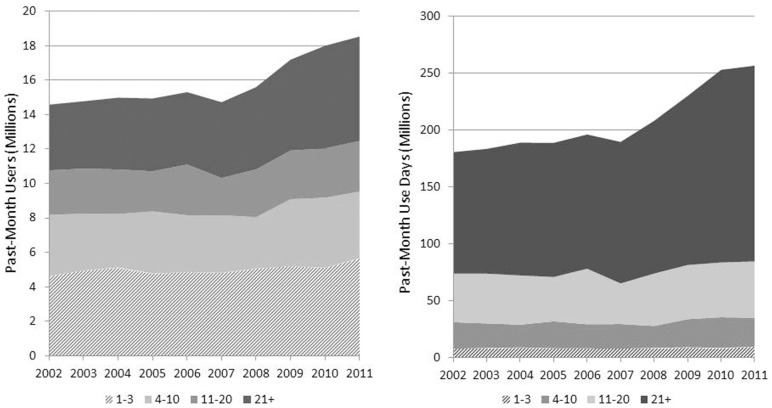
**Daily/near-daily users (21 days or more in the past-month) represent a minority of users yet are responsible for the majority of past-month use-days**. Note: because NSDUH did not collect data about blunts in 2002 and 2003, use-days may be underestimated for these years.

We calculated the total number of past-month use-days for each year from 2002 through 2011 and divide this total across four frequency of use categories: those who used 1–3 days, those who used 4–10 days, those who used 11–20 days, and those who used 21 days or more in the last month (daily/near-daily users). Figure 3 shows the growth in the total number of users and total number of use-days for all four groups. Although daily/near-daily users represented less than one-quarter of past-month cannabis users in 2002 and roughly one-third of past-month users in 2011, they account for the vast majority of use-days and are thus are presumably responsible for the majority of consumption.

**Figure 4:**
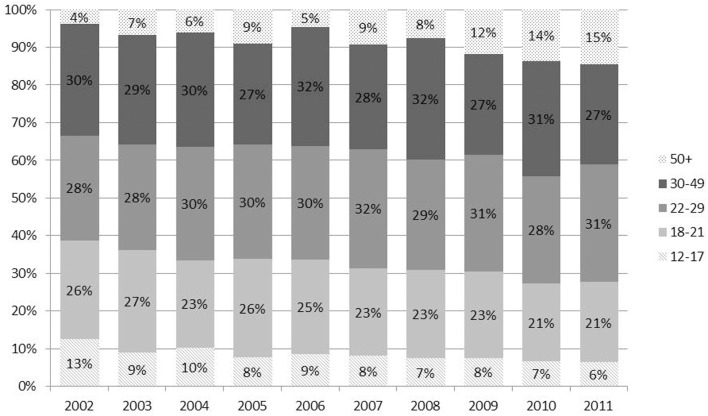
**Age distribution of daily near-daily cannabis users shifts over time so that older adults are responsible for an increasing proportion of consumption**. Note: because NSDUH did not collect data about blunts in 2002 and 2003, use-days may be underestimated for these years.

To understand more about the daily/near-daily users who are driving the increase in consumption, we explored their demographic characteristics over this 10-year time period. Examining the age distribution of the daily/near-daily users shows that youth’s share of consumption plummeted by almost 50%, and more generally consumption shifted to older adults (see Figure 4). In 2002, 12–17-year-olds represented 13% of daily/near-daily users; in 2011, that had dwindled to 7%. The proportion of daily/near-daily users attributable to young adults (ages 18–21 years) also decreased from 26% in 2002 to 21% in 2011. The proportion aged 22 years and older increased from 62 to 73%. In other words, the age distribution of daily/near-daily users shifted so that the average age of daily/near-daily users is higher in 2011 than it was in 2002.

There was a notable inversion of the ratio of youth (ages 12–17) to older adults (ages 50 and up). In 2002, there were more than three times as many youth as older adults using cannabis on a daily/near-daily basis; in 2011 there were 2.5 times more older adults than youth using on a daily/near-daily basis.

We found a similar shift in the age distribution of daily/near-daily users of alcohol and cigarettes; however, it was not as dramatic and we did not see the same inversion that was observed for cannabis use (see Table 3). There was disproportionate growth in older populations over this time due to the aging of the “baby boom” generation (14, 15), which explains most of the growth in older daily/near-daily users of alcohol and most of the growth in older daily/near-daily users of cigarettes. However, the increase in the proportion of older daily/near-daily users of cannabis was much greater than the increase in the proportion of older individuals in the population, suggesting an increase in heavy cannabis use among older individuals.

**Table 3:** **Change in demographic profiles of daily/near-daily users of cannabis, cigarettes, and alcohol, 2002–2011**.

	Cannabis (%)			Cigarettes (%)		Alcohol (%)	
	2002	2004	2011	2002	2011	2002	2011
**AGE (AMONG ALL USERS)**							
12–17	13	11	6	3	2	1	0
18–21	26	25	21	9	7	4	2
22–29	28	32	31	16	18	8	9
30–49	30	32	27	46	40	35	29
50+	4	7	15	25	33	53	60
**RACE/ETHNICITY (AMONG ALL USERS)**							
Non-Hispanic white	75	73	66	79	77	85	88
Non-Hispanic black	14	13	16	10	10	7	5
Hispanic	8	10	14	7	8	6	5
Other	3	4	4	5	6	2	3
**EDUCATION (AMONG ADULTS)**							
Less than high school	25	25	22	22	21	14	8
High school graduate	34	34	37	41	39	29	25
Some college	32	27	29	26	28	25	24
College graduate	9	13	12	11	13	32	43

Because NSDUH did not collect data about blunts in 2002 and 2003, use-days may be underestimated for these years.

We also examined the distribution of race/ethnicity and found an increase in the proportion of Hispanic daily/near-daily cannabis users (from 8% in 2002 to 14% in 2011). While the proportion of the population identifying as Hispanic increased over this time period (16, 17), the relative increase in the population was not as large as the relative increase in daily/near-daily cannabis users. We also found a decrease in the proportion of non-Hispanic white daily/near-daily cannabis users (from 75% in 2002 to 66% in 2011) and little change in the proportion of non-Hispanic black daily/near-daily users, who represented 14% of daily/near-daily cannabis users in 2002 and 16% in 2011. There was not a parallel change in the distribution of race/ethnicity among daily/near-daily users of cigarettes or alcohol (see Table 3).

Educational attainment was relatively stable from 2002 to 2011 for daily/near-daily users of cannabis and cigarettes, but there was a shift in the proportion of daily/near-daily alcohol users, so that the group was more educated at the end of the time period (86% had more than a high school education in 2002, while 92% had more than high school education in 2011 – see Table 3).

#### Past-month use-days

**Figure 5:**
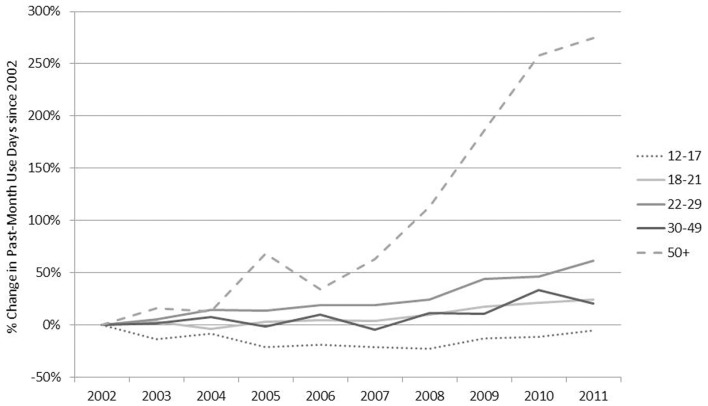
**Past-month use-days among older adults (50 and over) increased dramatically over this 10-year time period while use-days among youth (12–17) remained fairly stable**. Note: because NSDUH did not collect data about blunts in 2002 and 2003, use-days may be underestimated for these years.

For the most part, demographic changes in daily/near-daily users are also reflected in past-month use day trends. We explored changes in the past-month use-days since 2002 and found that consumption among adults over 50 grew sharply over the past 10 years while past-month use-days among those less than 18 years of age remained relatively stable (see Figure 5 – note that base rates for older users in 2002 are relatively low).

From 2002 to 2011, all race/ethnicities experienced growth in the number of cannabis use-days, particularly after 2008. Hispanics and other races had the largest relative increases (130 and 105%, respectively); however, despite the relatively slower growth, non-Hispanic white users continue to be responsible for the majority of use-days (see Table 4).

**Table 4:** **Cannabis past-month use-days (millions) by demographic groups over time**.

	Use-days (millions)		
	2002	2004	2011
**AGE (AMONG ALL USERS)**			
12–17	23.2	21.3	21.9
18–21	44.0	42.1	54.6
22–29	47.5	54.2	76.5
30–49	56.6	60.7	68.1
50+	9.5	10.7	35.6
**RACE/ETHNICITY (AMONG ALL USERS)**			
Non-Hispanic white	133.0	134.6	169.7
Non-Hispanic black	26.2	27.2	39.2
Hispanic	15.4	19.9	35.3
Other	6.1	7.3	12.4
**EDUCATION (AMONG ADULTS)**			
Less than high school	36.4	37.9	48.9
High school graduate	53.4	57.6	83.8
Some college	48.6	48.4	69.0
College graduate	19.1	24.0	33.0

Because NSDUH did not collect data about blunts in 2002 and 2003, use-days may be underestimated for these years. We have included data from 2004 when the questions about blunts were introduced for reference.

An exploration of use-days by education level shows less dramatic change than the change in age distribution (see Table 4). There was growth in the number of use-days among all adults regardless of education level; overall, use-days among adults increased by 49% over this time period. However, the largest relative increase was among those with a college degree, whose use-days increased by 72% from 2002 to 2011.

**Figure 6:**
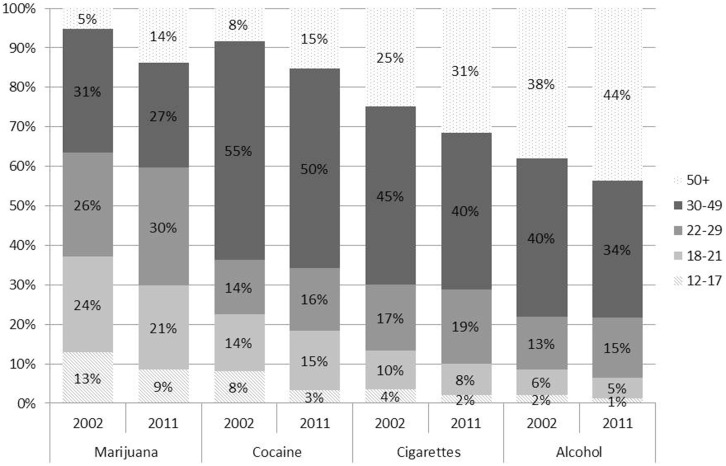
**A greater proportion of cannabis, alcohol, cigarettes, and cocaine use-days are attributable to older adults in 2011**. Note: because NSDUH did not collect data about blunts in 2002 and 2003, cannabis use-days may be underestimated for these years.

The shift in the distribution of use-days and daily/near-daily users from a younger to an older population is noteworthy. For comparison, we display the age distribution of alcohol, cigarettes, and cocaine use-days to determine whether a similar shift occurred with other substances (see Figure 6). Adults over the age of 30 were already responsible for the majority of alcohol, cigarettes, and cocaine use-days in 2002; however, there was a similar shift in the percent of cocaine, cigarette, and alcohol use-days attributable to those older than 50 and away from those 21 and under.

### Contrasting use-days with amounts used

Although use-days provide more information about consumption than does prevalence alone, weighting respondents by days of use may still understate the skew in the distribution of use. This is due to an apparent positive correlation between *intensity* of use (grams consumed per day) and the *frequency* of use (days of consumption per month). Zeisser et al. (7), for example, observe a positive correlation between the reported number of joints consumed per day and self-reported days of use per month. Their data suggest that those using on 30 days per month consumed about three times as many joints per day as did those using only 1–4 days per month.

**Figure 7:**
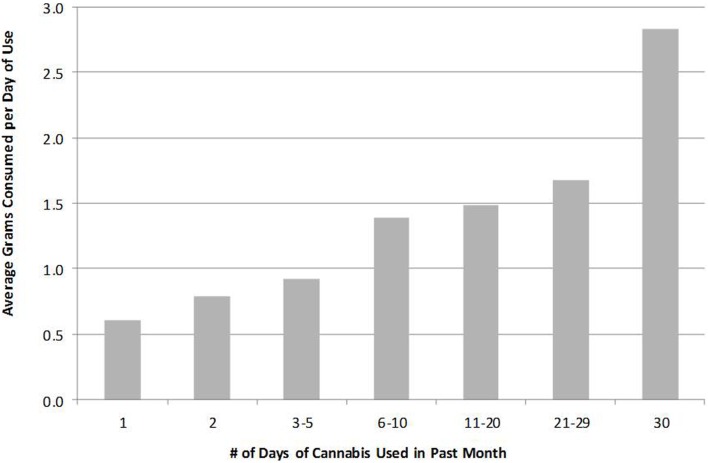
**Average quantity of cannabis consumed per day increases with frequency of cannabis use**.

Zeisser and colleagues’ analysis does not consider the possibility that joint or unit *size* might also be positively correlated with frequency of use, but the EUMII web-survey (4) described above did gather information about quantity consumed per use-day (in grams) by using picture cards. The EUMII data suggest that when denominating by quantity (weight) consumed instead of number of units, that ratio may be closer to 4:1 (see Figure 7).

This relationship has important implications for what one might term “equivalence ratios.” Naturally it takes multiple light users to consume as much as one heavy user, but how many? That depends on the measure of use. In particular, since it appears that those who use frequently also consume more per day of use, the ratios are considerably higher when the equivalence is one in terms of units or grams used rather than days of use.

**Figure 8:**
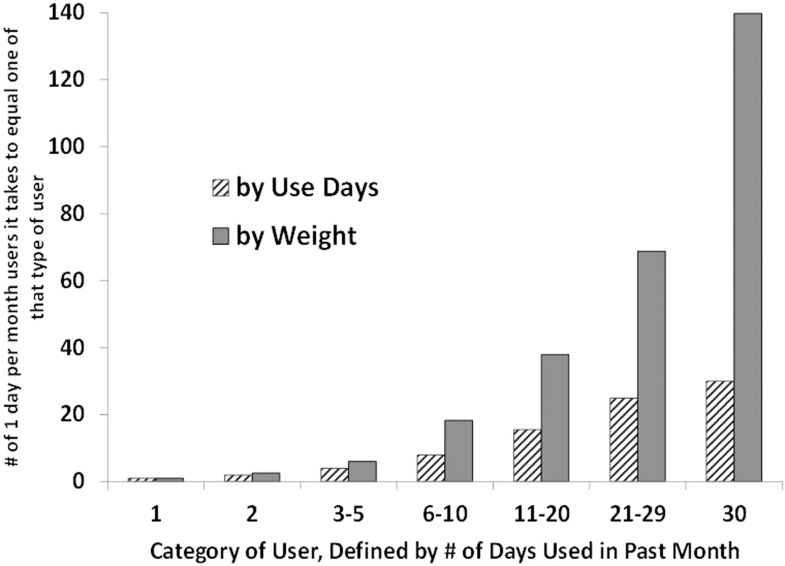
**The number of 1 day per month users required to match usage of more frequent users is higher when measuring usage in terms of grams per month rather than past-month use-days**.

If one focuses on days of use, it would take about 10–12 people using 1–5 times per month to match one daily user, but it would take more than three times that many ( >40) to match a single daily user in terms of grams consumed per month. Figure 8 shows these equivalence ratios for each of the categories of users, and with equivalence expressed in terms of both days of use (striped bars) and grams per month (solid bars).

Consider what this means for daily users’ share of the market. The one-in-five past-month users who consume daily account for almost 60% of consumption, while the one-third of past-month users who consume less than four times per month account for just 2% of consumption.

## Discussion

The best metric for studying cannabis clearly depends on the objective of the research. For those interested in the prevalence of cannabis use, the number of users is likely sufficient. However, to obtain a more accurate portrayal of cannabis use and users’ behavior or to better understand the market, one should look to frequency and amount of consumption. Likewise, those interested in drug-related criminal justice outcomes should focus on behavior that increases risk of arrest, such as the number of drug purchases and location of these purchases (e.g., indoor vs. outdoor).

Examining frequency of use over time provides a picture of not only changes in who is using but also how individuals are using. Beginning in 2007, there were increases not only in the number of users but also in the number of use-days per user and the number of daily/near-daily users, suggesting heavier use over this time period. Some may wonder if this increase might be attributable to more honest reporting about cannabis use. (One way to assess this would be to examine how support for legalization in the Gallup poll changed over this period, but the lack of poll data between 2005 and 2009 complicates this exercise (18)]. However, there are supply side indicators which suggest a large increase in domestic and Mexican production post-2005 [El Paso Intelligence Center (19)].

The demographic shifts in cannabis use-days and daily/near-daily users (particularly the shift from a younger to an older population) are intriguing and raise additional questions. Given our knowledge of drug use cycles and awareness that initiation of drug use typically happens at a young age (20, 21), can the increase in use among older individuals be attributed entirely to carrying drug use habits over time (which seems unlikely given the increase in use with respect to the relative increase in the older population) or something else? Are these older users using for medicinal or recreational purposes? Are these trends reflected in arrest or treatment datasets? Are users replacing cannabis use with use of another substance? Why did use-days among Hispanics increase so dramatically over this time period relative to other racial-ethnic groups? Does the increase in use-days among college-educated individuals indicate greater social acceptability or something else?

Zeisser et al. (7) and the EUMII web-survey (4) indicate that in Europe amount consumed per day is positively correlated with frequency of use, and thus, heavy users are responsible for a greater share of consumption than of days of use. A logical next question might be whether that pattern holds also for U.S. cannabis users and whether that means the average amount consumed per past-month user has increased along with frequency of consumption, at least in potency-adjusted terms. Preliminary analyses of data from Arrestee Drug Abuse Monitoring (ADAM) suggest there was not a statistically significant change in the average size of a joint over the 2000s (Kilmer et al., in preparation), but this is not a settled question. Further, future analyses must also account for the fact that cannabis is consumed in a variety of ways other than smoking joints (e.g., pipes, vaporizers, edibles) and that there may be substantial variation in potency as well.

In summary, by sampling on use-days and amount used, we find that most of the consumption and, hence, most of the associated intoxication and flow of money into the black markets, comes from people who use frequently. Examining the number of users can be enlightening but does not fully capture the dynamics of cannabis usage. In order to understand market-related quantities like demand, and to better assess implications for crime, health, and productivity, researchers should analyze cannabis usage indicators like use-days and quantity consumed.

## Conflict of Interest Statement

The authors declare that the research was conducted in the absence of any commercial or financial relationships that could be construed as a potential conflict of interest.
