# Erratum: Statistics on Cannabis Users Skew Perceptions of Cannabis Use

**DOI:** 10.3389/fpsyt.2013.00180

**Published:** 2013-12-24

**Authors:** Rachel Melissa Burns, Jonathan Paul Caulkins, Susan S. Everingham, Beau Kilmer

**Affiliations:** ^1^RAND Corporation, Pittsburgh, PA, USA; ^2^Carnegie Mellon University, Heinz College, Pittsburgh, PA, USA

The authors would like to submit a correction to Table 2 of the above article. The proportion of past-year users that are college graduates should be listed as 22 percent. The corrected table is printed here.

**Table 2:** **Past-year use, number of drug-related arrests, and number of monthly purchases by education level and racial-ethnic group, 2011 NSDUH**.

	Proportion of past-year users (%)	Proportion of those arrested for drug offenses (%)	Proportion of buys (%)
**(AMONG ADULTS)**			
Less than high school	16	23	27
High school graduate	31	39	37
Some college	32	34	30
College graduate	22	3	6
**(AMONG ALL USERS)**			
Non-hispanic white	67	53	55
Non-hispanic black/Afr Am	13	23	24
Hispanic	14	18	15
Other	6	7	6

